# An imbalanced ratio between PC(16:0/16:0) and LPC(16:0) revealed by lipidomics supports the role of the Lands cycle in ischemic brain injury

**DOI:** 10.1074/jbc.RA120.016565

**Published:** 2020-12-10

**Authors:** Lifeng Zheng, Chengbin Xie, Ju Zheng, Qiangrui Dong, Tengxiao Si, Jing Zhang, Sheng-Tao Hou

**Affiliations:** 1 Brain Research Centre and Department of Biology, Southern University of Science and Technology, Shenzhen, Guangdong Province, China; 2 Department of Biochemistry, Microbiology, and Immunology, Faculty of Medicine, University of Ottawa, Ottawa, Ontario, Canada

**Keywords:** cerebral ischemia, lipidomics, UPLC-MS/MS, PC(16:0/16:0), LPC(16:0), neurite outgrowth, growth cone, microglia, ChE, Cholesteryl ester, CL, cardiolipin, DG, diglyceride, DMEM, Dulbecco’s Modified Eagle Medium, dMePE, dimethylphosphatidylethanolamine, LPC, lysophosphatidylcholine, LPE, lysophosphatidylethanolamine, MCAO, middle cerebral artery occlusion, MRM, multiple reaction monitoring, OPLS-DA, orthogonal partial least squares discriminant analysis, PC, phosphatidylcholine, PCA, principal component analysis, PE, phosphatidylethanolamine, PG, phosphatidylglycerol, PI, phosphatidylinositol, PS, phosphatidylserine, SIMCA, soft independent modeling of class analogy, SM, sphingomyelin, TG, triglyceride, VIP, variable importance for the projection

## Abstract

Promoting brain recovery after stroke is challenging as a plethora of inhibitory molecules are produced in the brain preventing it from full healing. Moreover, the full scope of inhibitory molecules produced is not well understood. Here, using a high-sensitivity UPLC-MS-based shotgun lipidomics strategy, we semiquantitively measured the differential lipid contents in the mouse cerebral cortex recovering from a transient middle cerebral artery occlusion (MCAO). The lipidomic data were interrogated using the soft independent modeling of class analogy (SIMCA) method involving principal component analysis (PCA) and orthogonal partial least squares discriminant analysis (OPLS-DA). Statistics of the 578 confirmed lipids revealed 84 species were differentially changed during MCAO/reperfusion. The most dynamic changes in lipids occurred between 1 and 7 days post-MCAO, whereas concentrations had subsided to the Sham group level at 14 and 28 days post-MCAO. Quantitative analyses revealed a strong monotonic relationship between the reduction in phosphatidylcholine (PC)(16:0/16:0) and the increase in lysophosphatidylcholine (LPC)(16:0) levels (Spearman’s Rs = −0.86) during the 1 to 7 days reperfusion period. Inhibition of cPLA2 prevented changes in the ratio between PC(16:0/16:0) and LPC(16:0), indicating altered Land’s cycle of PC. A series of *in vitro* studies showed that LPC(16:0), but not PC(16:0/16:0), was detrimental to the integrity of neuronal growth cones and neuronal viability through evoking intracellular calcium influx. In contrast, PC(16:0/16:0) significantly suppressed microglial secretion of IL-1β and TNF-α, limiting neuroinflammation pathways. Together, these data support the role of the imbalanced ratio between PC(16:0/16:0) and LPC(16:0), maintained by Lands’ cycle, in neuronal damage and microglia-mediated inflammatory response during ischemic recovery.

Cerebral ischemia is caused by the transient or permanent occlusion of blow flow to the brain, which leads to severe brain damage and long-term functional impairments (1, 2). Reduction in blood flow lowers energy supply, disturbs membrane ionic balance, depolarizes the neuronal membrane leading to the excessive release of excitatory neurotransmitters such as glutamate (3, 4, 5). Glutamate-receptor-mediated elevation in intracellular Ca^2+^ concentrations activates calcium-dependent proteases, which break down critical structural proteins leading to active neuronal death with morphological features present on the continuum between typical necrosis and apoptosis (4, 6). Brain function recovery is challenging and often incomplete after cerebral ischemia. One of the main reasons for the limited regeneration in the injured brain is due to the production of a plethora of inhibitory lipids in the damaged territory (7, 8, 9, 10, 11, 12, 13). Indeed, blood lipids are strong indicators for ischemic and hemorrhagic stroke in patients (14). Understanding dynamic lipid changes during ischemic brain recovery will reveal novel mechanisms in nerve regeneration, brain repair, and shed light on developing potential therapeutics.

Membrane phospholipids are fluidic structures that can respond to external environments through changes in their fatty acid composition and density on cell membranes. Lipids are in constant flux and are continuously converted into each other. For example, phosphatidylcholine (PC) is a major component of the biological membrane accounting for ∼40 to 50% of total phospholipids (15). The fatty acyl moieties of membrane phospholipids exhibit considerable diversity in chain length and degree of saturation.

Removing PC fatty acid chains at the *sn*-2 position *via* the action of cytosolic phospholipase A2 (cPLA2) results in the formation of lysophosphatidylcholine (LPC) (13, 16). LPC can be converted back to PC by the enzyme LPC acyltransferase in the presence of Acyl-CoA (17). The dynamic changes in the content ratio of PC-LPC represent the body’s *de novo* cyclical synthesis and degradation of PC as part of the Lands cycle (16, 17, 18).

The level of LPC is altered in the brain following both focal and global cerebral ischemia in rats and mice (8, 12, 19, 20, 21). A transient increase of LPC concentrations up to 200 μM was reported in the brain of a rat model of cerebral ischemia (22, 23), suggesting a fast turnover of LPC in ischemic brains. Furthermore, the PC-LPC balance maintained by the Lands cycle appears essential. Indeed, the deletion of cPLA2, the enzyme for converting PC to LPC, is neuroprotective against cerebral ischemia (24, 25). Inhibition of cPLA2 alpha by a monoclonal antibody attenuated focal cerebral ischemic in mice (26). However, the specific functions of PC and LPC in ischemic brains reported in the literature are conflicting with either protective or detrimental effects. Nevertheless, it has been shown that hyperoxia preconditioning significantly decreased the brain LPC levels and ischemic brain injury (27), suggesting an important association of LPC content with brain injury. Excessive conversion of PC to LPC appears to be detrimental to the brain recovering from cerebral ischemia.

Numerous studies have shown ischemic brain injury is associated with microglial activation (7, 28, 29, 30, 31). Microglia are the primary source of IL-1β in the brain (32), and LPC, in general, stimulates IL-1β release from microglia (33). However, the role of LPC(16:0) and PC(16:0/16:0) in microglia-mediated neuroinflammation is unclear. Administration of lipopolysaccharides (LPS) also results in dramatic IL-1β expression by microglia, serving as an excellent model to simulate microglia response in the ischemic brain (34).

To identify lipid changes in ischemic brains, we used a well-characterized mouse long-term recovery model with a 30 min transient occlusion of the middle cerebral artery (MCAO), followed by up to 28 day long-term reperfusion. This MCAO model is a widely used mouse model simulating transient ischemic attacks in the clinics. Of the 578 lipid species identified in the ischemic brain, 84 of them changed significantly, which belonged to 16 lipid groups. Notably, the PC(16:0/16:0)-LPC(16:0) ratio changed dramatically during the ischemic reperfusion suggesting the impairment of Lands’ cycle. Series of *in vitro* experiments were performed to demonstrate the role of PC and LPC in neurite outgrowth, neuronal viability, and microglia-mediated inflammatory response.

## Results

### Long-term transient mouse MCAO model and the lipidomic profiling workflow

**Figure 1:**
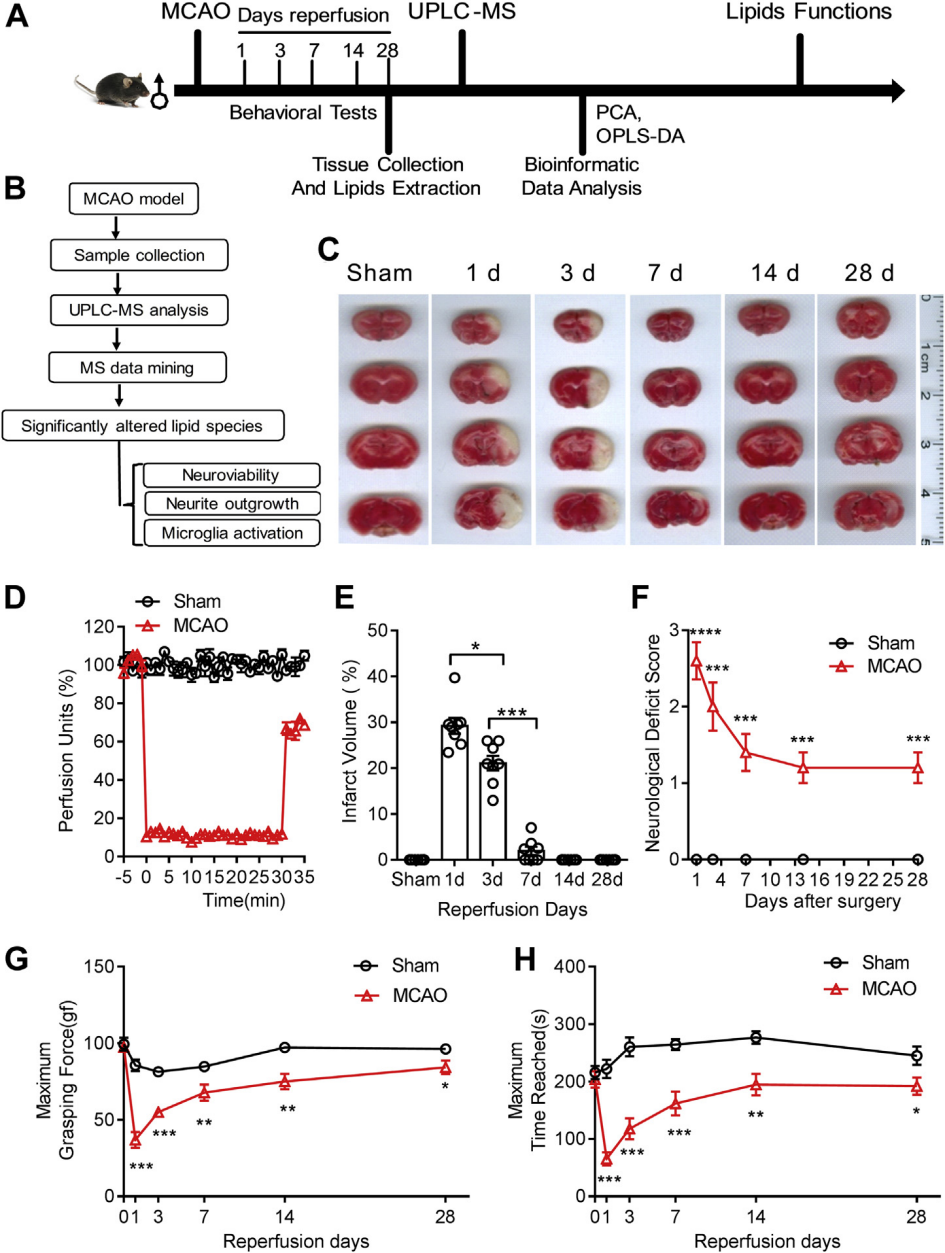
**Transient mouse MCAO/reperfusion model and the lipidomic profiling workflow.***A*, a schema of the time course to generate the MCAO model, tissue analysis, and lipid function studies. *B*, a schema illustrates the workflow to obtain lipid metabolites and their functions. *C*, TTC staining of coronal brain sections at 2 mm thickness to show ischemic (*white-colored* on the right hemisphere) and the contralateral side (*red-colored tissue* on the left hemisphere). *D*, Laser Doppler flowmetry measurements of blood flow of the middle cerebral artery before and after MCAO. *E*, the infarct volume measured from the TTC stained brain coronal sections. *F*, neurological deficits scores were measured from MCAO and Sham group of mice for up to 28 days reperfusion. The forepaw pulling strength test (*G*) and rotor rod performance test (*H*) from the Sham and MCAO groups. Data represent the mean ± SD. Error bars indicate SD. n = 8, ∗ indicates *p* < 0.05, ∗∗*p* < 0.01, ∗∗∗*p* < 0.001 by one-way ANOVA with LSD *post hoc* analysis to identify significant groups when compared between MCAO with the Sham group.

As illustrated in Figure 1*A*, male mice at a bodyweight of 21 to 23 g with age between 6 and 8 weeks were randomly divided into two groups to receive sham operation or transient MCAO surgery. The MCAO model was the most widely used thread-based transient MCAO model, which recapitulates the transient ischemic attacks in the clinics. The model is carefully characterized as follows to provide a standardized platform for future research comparisons and identification of druggable targets (Fig. 1, *B*–*H*). After 30 min MCAO, the silicon-coated thread was withdrawn from the external carotid artery to allow reperfusion monitored using Doppler flow cytometry (Fig. 1*D*). Mice were allowed to recover for up to 28 days. TTC staining of coronal brain sections showed ischemic infarction in the cerebral cortex close to the middle cerebral artery and part of the striatum (Fig. 1*C*), affecting both motor and sensory cortices of the brain. The volume of ischemic infarction increased significantly in 1- and 3-day groups and decreased to the Sham group level in the 14 and 28 day post-MCAO groups (Fig. 1*E*). Correspondingly, the neurological deficits (Fig. 1*F*), forepaw pulling strength (Fig. 1*G*), and rotarod balance test (Fig. 1*H*) also showed a significant deterioration at 1 to 7 days post-MCAO. Although the levels of these motor functions in the MCAO group recovered gradually, they could not return to the exact Sham group level at 14 days and 28 days post-MCAO (*p* < 0.05), indicating an incomplete functional rehabilitation process.

### Lipidomic profiling features from MCAO mouse cortex

**Figure 2:**
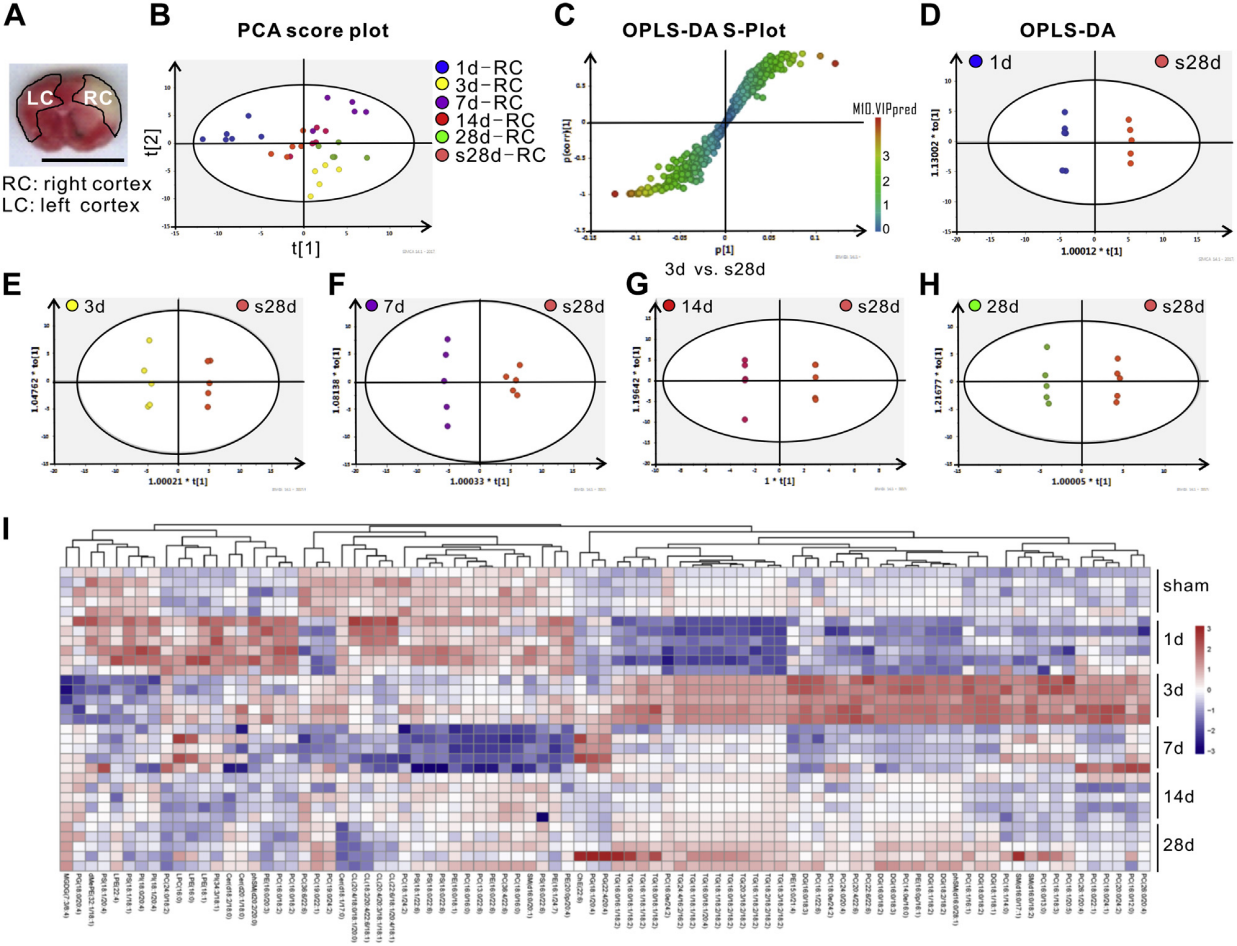
**Lipidomic profiling analysis.***A*, an image of a coronal section of the ischemic mouse brain taken from Figure 1*C* (second column/second row), as a schema just to illustrate RC and LC locations collected for lipidomic profiling. The scale bar = 1 cm. Lipid features data were analyzed using pattern recognition models and statistics, including PCA scatter plot (*B*), S-Plot (*C*) OPLS-DA (*D*–*H*). *I*, the heatmap generated from the 84 differentially expressed lipids was produced using the corresponding data from every mouse in the group (n = 5 per group).

To fully understand lipid changes in the injured cortices, mouse left (LC) and right (RC) cortices in the ischemic territory of mice at 1, 3, 7, 14, and 28 days post-MCAO were dissected out and underwent lipid isolation and UPLC-MS/MS analysis (Fig. 2*A*). Lipid features data were collected, confirmed through comparison with the LipidSearch database, and analyzed using pattern recognition models and statistics, including principal component analysis (PCA), OPLS-DA, S-Plot, and Student’s *t*-test. Based on the specific fragment patterns and retention times, we ultimately identified a total of 578 lipids. Of those, 84 metabolites were significantly and differentially expressed between the Sham and MCAO groups (Table 1).

**Table 1:** List of 84 lipid species differentially expressed in the right cortex of mouse brain after MCAO throughout the 28 days of reperfusion

Lipids groups	Lipids species
Ceramide	Cer(d18:1/17:0), Cer(d18:2/18:0), Cer(d20:1/18:0)
Cholesteryl ester	ChE(22:6)
Cardiolipin	CL(18:2/20:4/22:6/18:1), CL(20:4/18:0/18:1/20:0), CL(20:4/20:3/18:1/18:1), CL(22:6/18:1/20:4/18:1)
Diglyceride	DG(16:0/18:2), DG(16:0/18:3), DG(16:0/18:3), DG(18:0/18:2), DG(18:1/18:1), DG(18:1/18:2), DG(18:2/18:2)
Triglyceride	TG(16:0/16:1/18:2), TG(16:0/18:1/18:2), TG(16:0/18:1/20:4), TG(16:0/18:2/18:2), TG(16:1/18:1/18:2), TG(16:1/18:2/18:2), TG(18:0/18:1/18:2), TG(18:1/18:1/18:2), TG(18:1/18:2/18:2), TG(18:2/18:2/18:2), TG(18:3/18:2/18:2), TG(20:3/18:2/18:2), TG(24:4/16:2/16:2)
Lysophosphatidylcholine	LPC(16:0)
Lysophosphatidylethanolamine	LPE(16:0), LPE(18:1), LPE(22:4)
Dimethylphosphatidylethanolamine	dMePE(32:1/18:1)
Phosphatidylcholine	PC(13:0/22:6), PC(14:0e/16:0), PC(16:0/12:0), PC(16:0/13:0), PC(16:0/16:0), PC(16:0/18:2), PC(16:0/18:2), PC(16:0e/24:2), PC(16:1/14:0), PC(16:1/16:1), PC(16:1/18:3), PC(16:1/20:5), PC(16:1/22:6), PC(18:0/16:0), PC(18:0/22:1), PC(18:0/24:1), PC(18:0e/24:2), PC(18:1/24:2), PC(19:0/22:1), PC(19:0/24:2), PC(20:0/24:2), PC(20:4/22:6), PC(22:6/22:6), PC(24:0/18:2), PC(24:0/20:4), PC(26:0/20:4), PC(26:1/20:4), PC(36:4/22:6), PC(36:6/22:6)
Phosphatidylethanolamine	PE(15:0/21:4), PE(16:0/18:1), PE(16:0/20:3), PE(16:0/22:6), PE(16:0p/16:1), PE(16:1/24:7), PE(20:0p/20:4)
Phosphatidylserine	PS(16:0/22:6), PS(18:0/22:6), PS(18:0/22:6), PS(18:1/18:1), PS(18:1/20:4), PS(18:1/22:6)
Phosphatidylglycerol	PG(18:0/20:4), PG(18:1/20:4), PG(22:4/20:4)
Phosphatidylinositol	PI(18:0/20:4), PI(18:1/20:4), PI(34:3/18:1)
Sphingomyelin	SM(d16:0/17:1), SM(d16:0/18:2), SM(d16:0/20:1)
Sphingomyelin (phytosphingosine)	phSM(d16:0/28:1), phSM(d20:2/20:0)

Unsupervised PCA with QC samples was performed to assess the experiment quality. The PCA score scatter plots for the positive (Fig. 2*B*) and negative (not shown) ionization mode showed apparent differences in grouping between each MCAO group and the Sham group (labeled as s28days-RC in Fig. 2*B*) and a high degree of clustering within each group (indicated by the same colored dots within the circle).

As shown in Figure 2*C*, the supervised OPLS-DA S-plot showed evident lipid species influence on group separation, and the lipid species closer to the ends of S indicated a more significant difference between groups as exemplified by the comparison between 3-days MCAO and Sham group (Fig. 2*C*). Supervised OPLA-DA analysis was set at variable importance for the projection (VIP) > 1.5 with *t*-test (*p* < 0.05) and false discovery rate <0.01, which allowed the identification of 84 differentially expressed lipids during recovery from MCAO. OPLS-DA score scatter plots derived from the positive ionization mode were compared between groups shown in Figure 2, *D*–*H*. The 1, 3, 7, 14, and 28-day post-MCAO groups (blue, yellow, purple, red, and green colored dots, respectively) showed a clear separation for the Sham group (orange-colored dots) (Fig. 2, *D*–*H*). Furthermore, the normalized ratio of differential levels of the 84 lipid species showed an apparent clustering on the heatmap (Fig. 2*I*).

### Quantitative analyses of differentially altered lipids in the ischemic brain

The identified 84 differentially expressed lipids belonged to 15 lipid groups (Table 1), including Ceramide (Cer), Cholesteryl ester (ChE), Cardiolipin (CL), Diglyceride (DG), Triglyceride (TG), Lysophosphatidylcholine (LPC), Lysophosphatidylethanolamine (LPE), Dimethylphosphatidylethanolamine (dMePE), Phosphatidylcholine (PC), Phosphatidylethanolamine (PE), Phosphatidylserine (PS), Phosphatidylglycerol (PG), Phosphatidylinositol (PI), Sphingomyelin (SM), and Sphingomyelin-containing phytosphingosine (phSM).

**Figure 3:**
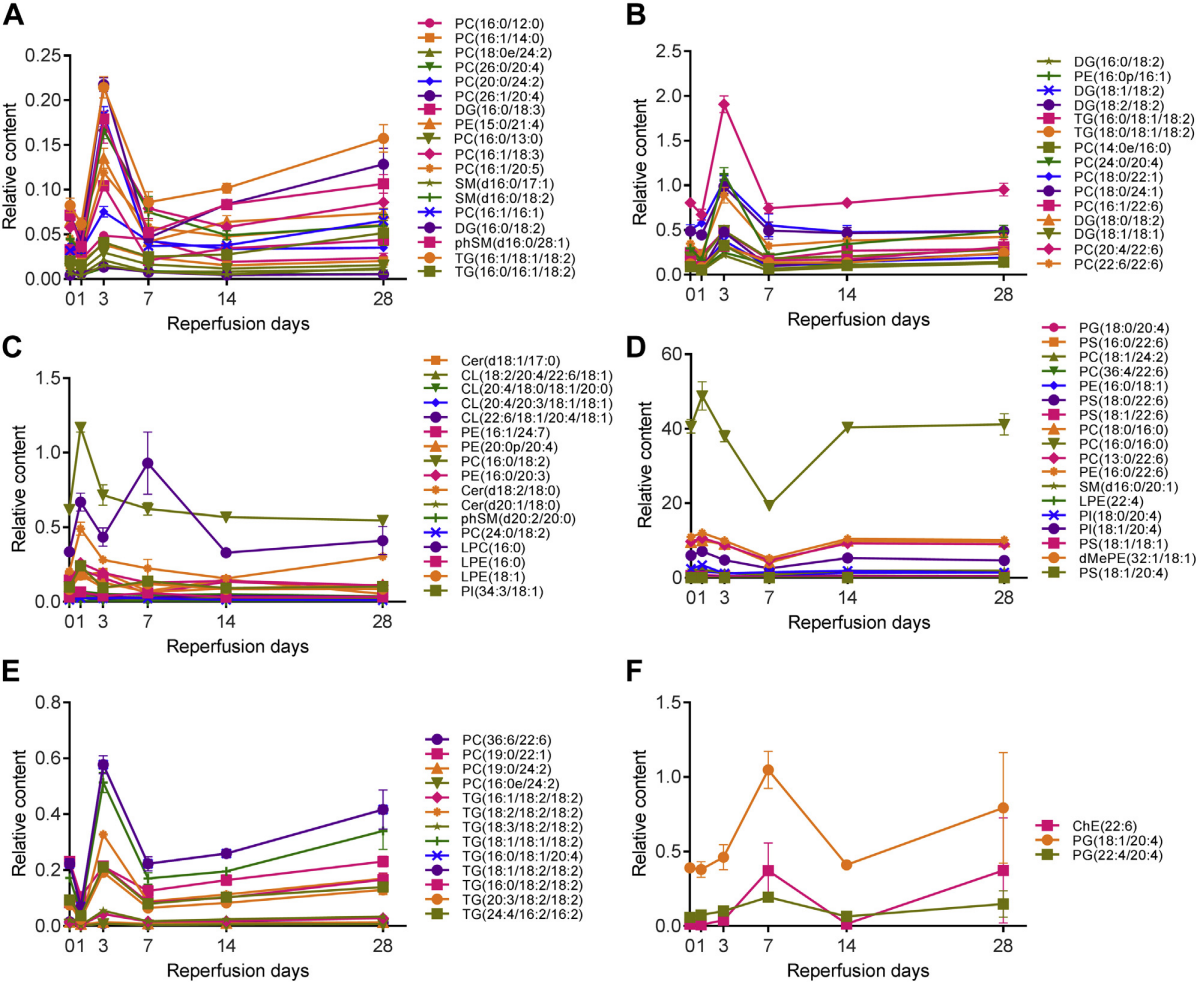
**Semiquantification of differentially expressed lipids in the ischemic brain.** The relative contents of all lipids were determined using Xcalibur 4.0 with normalization to that of the internal spiking control SM(d18:1/12:0). The relative content levels of the 84 differentially expressed lipids in the MCAO brain was plotted in *A*–*F* to show changed lipids between 1 and 28 days post-MCAO. Due to the large disparity in the relative contents, even within the same groups of lipids, it was impossible to plot lipids from the same group together. The lipid contents changed the most between 1 and 7 days and subsided between 14 and 28 days post-MCAO.

The relative contents of all lipids were determined using Xcalibur 4.0 to calculate the area of a peak in the chromatogram in multiple reaction monitoring (MRM) mode for the individual compounds. The area of the peaks of these MRM chromatograms was used for quantification with normalization to that of the internal spiking control SM(d18:1/12:0). The relative content levels of the 84 differentially expressed lipids were shown in Figure 3, *A*–*F*. The most dynamic changes in lipids profiles occurred between 1 and 7 days after MCAO. Most of the lipids returned to the Sham brain level at 14 to 28 day groups. In general, the levels of PI(18:0/20:4), PI(18:1/20:4), LPE(18:1), LPE(16:0), LPE(22:4), CL(20:4/18:0/18:1/20:0), CL(18:2/20:4/22:6/18:1), CL(22:6/18:1/20:4/18:1), CL(20:4/20:3/18:1/18:1), dMePE(32:1/18:1), phSM(d20:0/20:0) increased two to fourfolds within the 1 day of MCAO and returned to almost Sham level at the 14 to 28 days post-MCAO. Most of the phospholipids, including PC(16:1/20:5), PC(16:1/18:3), PC(16:0/13:0), PC(16:1/14:0), PC(16:0/12:0), PC(20:0/24:2), PC(18:0/24:1), PC(18:0/22:1), PC(26:1/20:4), PC(26:0/26:4), PC(16:1/16:1), PC(22:6/22:6), PC(24:0/20:4), PE(15:0/21:4), decreased after MCAO at 1 day, followed by an increase at 3 days post-MCAO. The content levels of PE(16:0/18:1), LPC(16:0), PG(22:4/20:4), PG(18:1/20:4), ChE(22:6) increased between 3 and 7 days post-MCAO. For most lipids, their content levels were back to the Sham group level at the 14 and 28 days post-MCAO.

**Figure 4:**
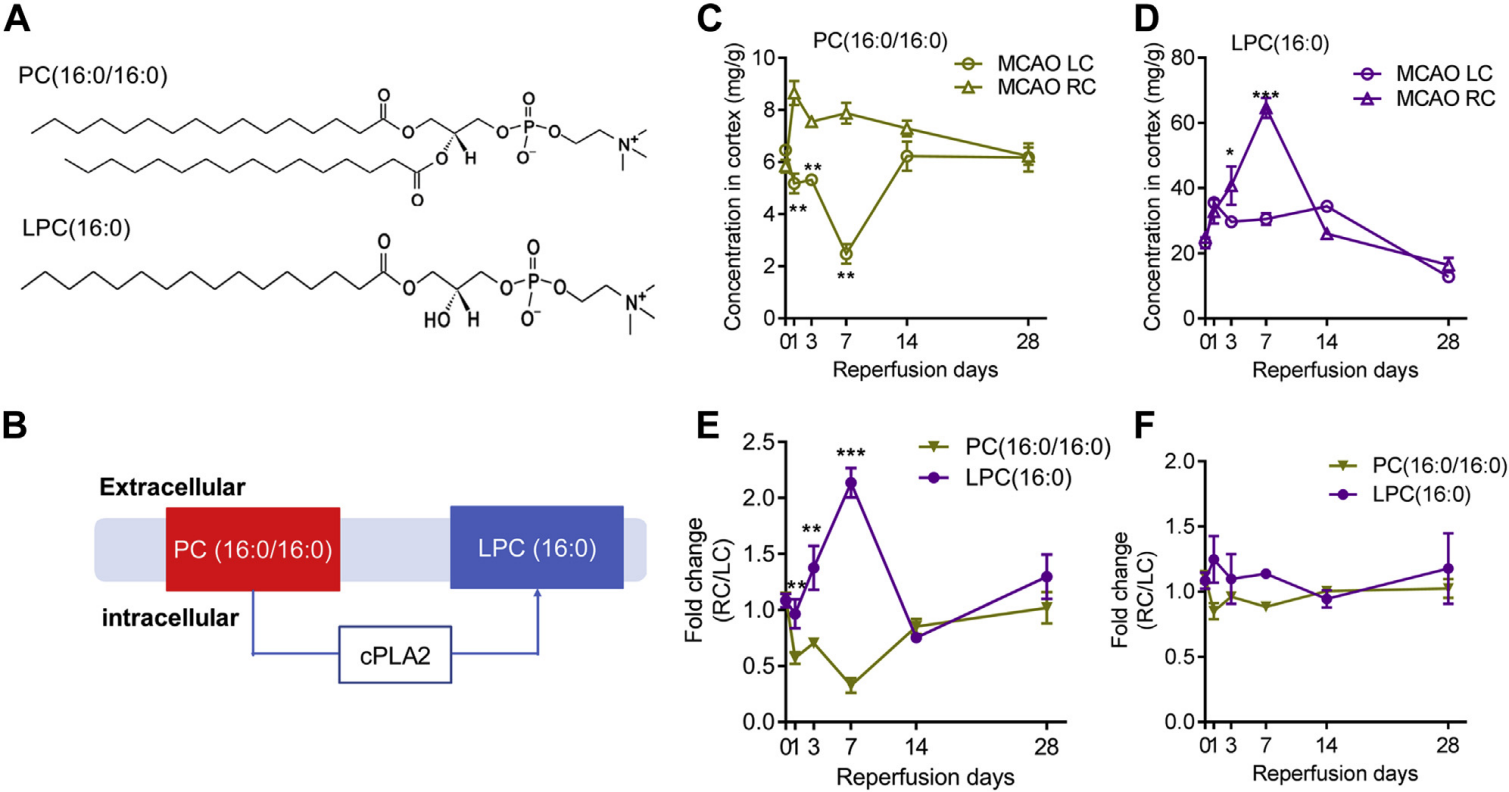
**Correlation between the levels of PC(16:0/16:0) and LPC(16:0) in the MCAO brain.***A*, showing the structures of PC(16:0/16:0) and LPC(16:0). *B*, a schematic diagram showing membrane-localized PC(16:0/16:0) to LPC(16:0) and the enzyme cPLA2 mediating the conversion of the two in the Lands cycle. Quantifications of PC(16:0/16:0) (*C*) and LPC(16:0) (*D*) in the MCAO brain left and right hemispheres (LC and RC, respectively) using the commercial lipid standards as internal controls. The fold changes (RC/LC) of PC(16:0/16:0) and LPC(16:0) were plotted in (*E*). *F*, the plot showing the fold changes (RC/LC) of PC(16:0/16:0) and LPC(16:0) from MCAO mice fed with ATK to inhibit cPLA2. Data represent the mean ± SD. Error bars indicate SD. n = 5, ∗ indicates *p* < 0.05, ∗∗*p* < 0.01, ∗∗∗*p* < 0.001 by one-way ANOVA with LSD *post hoc* analysis to identify significant groups when compared between MCAO with the Sham groups.

It was technically challenging to fully quantify all 84 lipids in the brain since the required synthetic lipid standards were not readily available. We were able to purchase pure lipid standards from Avanti Polar Lipids (Merck) for quantitative analysis of the content levels of six lipids (Table 2). The level of PE(16:0/18:1) decreased about threefold at 1 day post-MCAO, representing the highest reduction fold amongst all six quantified lipid species. But the content level of PE(16:0/18:1) increased over threefold at 3 days post-MCAO compared with that of the Sham brain, demonstrating the dynamic range of changes of PE(16:0/18:1). The basal level of Cer(d18:1/17:0) in the Sham brain is about 15 mg/g. Interestingly, the levels of Cer(d18:1/17:0) in both brain cortices increased 1 day post-MCAO to 45 mg/g on the contralateral side of the brain. The content levels of LPE(16:0) and LPE(18:1) were very low in the Sham animal brain at about 0.5 mg/g and 0.05 mg/g, respectively, and all increased at 3 days and 7 days post-MCAO. The content levels of PC(16:0/16:0) and LPC(16:0) were also increased significantly, shown in the next section, and Figure 4.

**Table 2:** Lipid contents in the MCAO brains

Lipid groups	PC(16:0/16:0)		LPC(16:0)		PE(16:0/18:1)		LPE(16:0)		LPE(18:1)		Cer(d18:1/17:0)	
	LC	RC	LC	RC	LC	RC	LC	RC	LC	RC	LC	RC
Sham	5.84 ± 0.55	6.47 ± 0.40	23.22 ± 3.80	24.90 ± 3.01	19.56 ± 2.03	22.20 ± 2.22	0.53 ± 0.05	0.61 ± 0.11	0.04 ± 0.01	0.07 ± 0.03	12.07 ± 3.11	15.77 ± 4.45
1 day	8.65 ± 1.13	5.20 ± 1.03	38.89 ± 6.61	32.82 ± 8.99	23.69 ± 2.10	8.80 ± 1.61	0.71 ± 0.13	0.44 ± 0.13	0.05 ± 0.02	0.08 ± 0.01	44.90 ± 4.32	22.31 ± 6.92
3 days	7.55 ± 0.47	5.32 ± 0.45	29.83 ± 2.67	40.80 ± 13.17	19.94 ± 2.20	26.84 ± 3.05	0.53 ± 0.10	0.77 ± 0.12	0.05 ± 0.01	0.08 ± 0.01	66.71 ± 7.03	29.71 ± 10.65
7 days	8.08 ± 0.91	8.28 ± 1.26	30.69 ± 4.64	61.09 ± 3.68	18.72 ± 3.56	19.40 ± 8.04	0.58 ± 0.07	0.67 ± 0.09	0.07 ± 0.02	0.13 ± 0.07	12.18 ± 7.69	13.30 ± 8.03
14 days	7.30 ± 0.68	6.23 ± 1.25	34.43 ± 2.02	26.01 ± 3.19	26.71 ± 2.06	17.66 ± 2.10	0.73 ± 0.09	0.54 ± 0.04	0.06 ± 0.01	0.07 ± 0.01	16.98 ± 2.86	13.89 ± 3.84
28 days	6.23 ± 0.75	6.18 ± 1.21	15.41 ± 5.52	24.59 ± 6.45	25.61 ± 1.62	26.13 ± 3.83	0.55 ± 0.10	0.65 ± 0.10	0.05 ± 0.01	0.06 ± 0.01	16.34 ± 2.79	13.45 ± 2.66

Data represents the average ± STDEV of n = 5 brains; unit = mg/g of brain tissue.

LC, contralateral side; RC, ipsilateral side.

### Strong monotonic relationship between the levels of PC(16:0/16:0) and LPC(16:0)

The content levels of PC(16:0/16/0) and LPC(16:0) in the right and left cortices of the MCAO brain were quantitated using specific lipid standards. The basal levels of PC(16:0/16/0) and LPC(16:0) in the Sham brains were at about 6 mg/g and 24 mg/g, respectively. As shown in Figure 4, *C*–*D*, the level of PC(16:0/16/0) decreased sharply at 1 day and 3 days post-MCAO, while LPC(16:0) level increased significantly at 3 days and 7 days post-MCAO, indicating an association of changes of the two during MCAO. The fold of change in the ipsilateral side (RC) from the contralateral side (LC) in PC(16:0/16:0) and LPC(16:0) demonstrated a sharp increase in LPC(16:0) and a dramatic reduction in PC(16:0/16:0) level at 1 day, 3 days, and 7 days post-MCAO (Fig. 4*E*).

The fact that only the level of LPC(16:0) changed post-MCAO significantly led us to hypothesize that there was an underlying relationship between PC(16:0/16:0) and LPC(16:0). To determine the relationship between changes in the levels of PC(16:0/16:0) and LPC(16:0), the Spearman correlation coefficient was calculated with *R*s = −0.86 at 1 to 7 days post-MCAO (*p* < 0.05), which demonstrated a robust monotonic correlation between the levels of PC(16:0/16:0) and LPC(16:0) during the early phase of MCAO. This association suggested that the increased LPC(16:0) was likely the result of an increased breakdown of PC(16:0/16:0).

Indeed, PC(16:0/16/0) is a substrate of cPLA2. cPLA2 mediates the removal of 16:0 fatty acid chain from PC(16:0/16:0) to produce LPC(16:0) (Fig. 4, *A*–*B*). The metabolic changes in the PC-LPC ratio may represent the body’s *de novo* cyclical synthesis and degradation of PC as part of the Lands cycle (17, 18). It is therefore highly possible that the increased LPC(16:0) in the ischemic brain was the result of the metabolic breakdown of PC(16:0/16:0).

To further demonstrate whether Lands’ cycle modulated the imbalance of the PC-LPC ratio during MCAO, cPLA2 inhibitor arachidonyl trifluoromethyl ketone (ATK) was gavage fed to mice 2 h after MCAO at 7.5 mg/kg body weight with carrier methylcellulose as previously described (35). The left and right cortices were assessed for PC(16:0/16:0) and LPC(16:0) contents post-MCAO. As shown in Figure 4*F*, the monotonic relationship between the fold changes of PC(16:0/16:0) and LPC(16:0) disappeared, providing additional evidence to support Lands’ cycle’s role mediating PC-LPC conversion during MCAO.

### LPC(16:0) causes growth cone collapse and neuronal death

**Figure 5:**
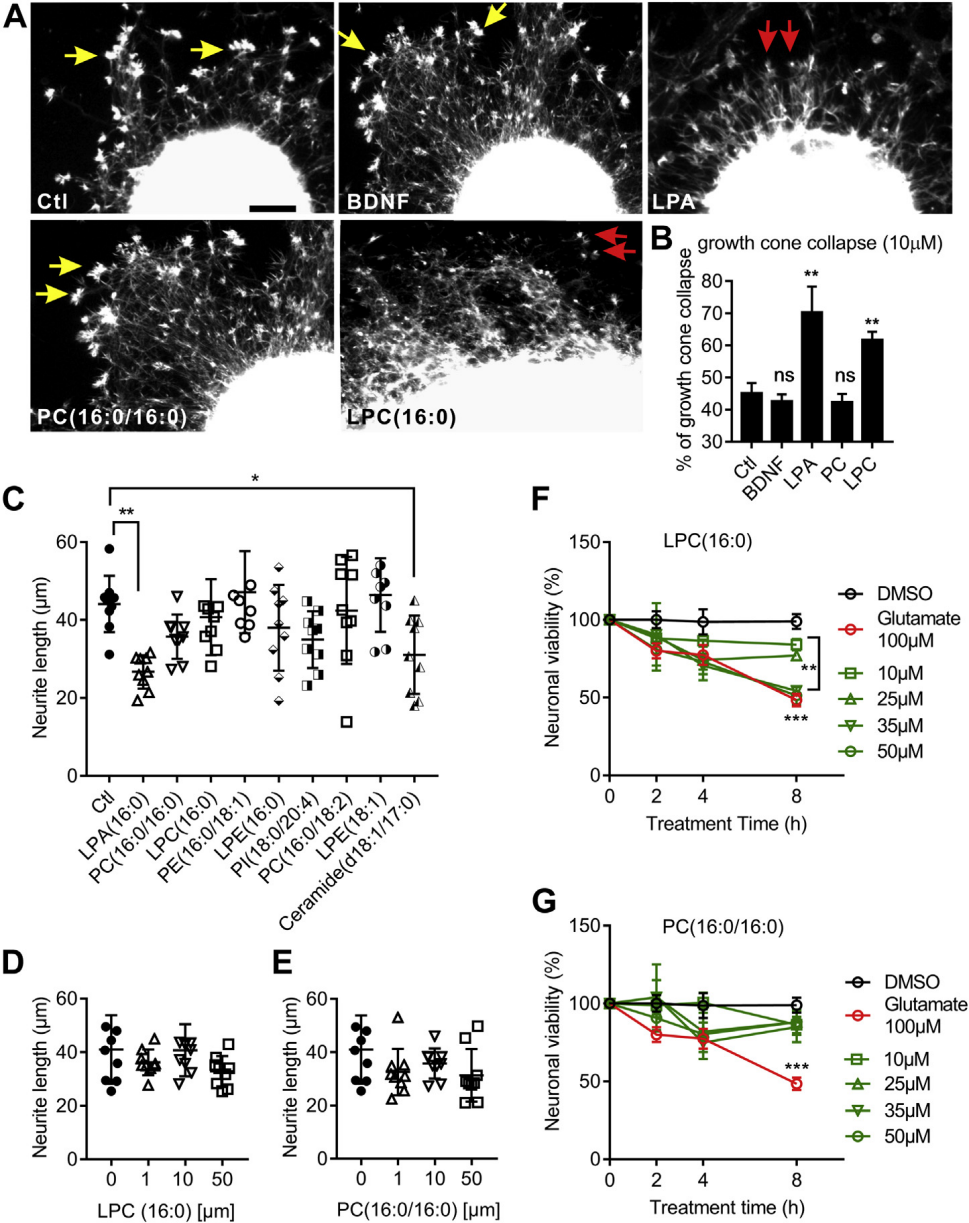
**LPC(16:0) causes growth cone collapse and neuronal death.***A*, eGFP transgenic mouse brain cortical explants were cultured for 3 days and treated with the agent as indicated on the microphotograph. Growth cones are indicated by yellow-colored arrows, and collapsed growth cones are shown by the red-colored arrows. The scale bar = 10 μm. *B*, the % of collapsed growth cones was counted and plotted (n = 10 independent explants per treatment). Neurite length of cortical explants treated with 10 μM of the indicated agent was measured using the Scholl method and plotted in *C*. The neurite lengths of cortical explants treated with LPC(16:0) (*D*) and PC(16:0/16:0) (*E*) at different doses were plotted. Cortical neuronal cultures treated with the indicated LPC(16:0) (*F*) and PC(16:0/16:0) (*G*) were examined for cell viability using the CCK-8 kit. DMSO and glutamate treatment were used as negative and positive controls, respectively, for neuronal death. Data represent the mean ± SD. Error bars indicate SD. n = 5 independent repeats, ∗ indicates *p* < 0.05, ∗∗*p* < 0.01, ∗∗∗*p* < 0.001 by one-way ANOVA with LSD *post hoc* analysis to identify significant groups.

Neurite outgrowth with fully extended growth cones is an essential feature of regenerating neurons forming networks (36). To further investigate the role of PC(16:0/16:0) and LPC(16:0) on neurons, cultured mouse cortical explants were used to determine their effects on growth cone integrity, neurite outgrowth, and cell viability (Fig. 5). After 3 days in culture, cortical explants showed extensive neurite extension and fully expended growth cones (Fig. 5*A*, yellow-colored arrows). Treatment with 10 μM lysophosphatidic acid (LPA) and LPC(16:0) caused significant growth cone collapse, while in contrast, treatment with BDNF and PC(16:0/16:0) did not induce growth cone collapse (Fig. 5, *A*–*B*).

The Sholl method (37) was used to determine PC(16:0/16:0) and LPC(16:0) effects on neurite outgrowth (Fig. 5, *C*–*E*). Treatment with 10 μM LPA and Cer(18:1/17:0) caused significant shortening of neurite extensions (Fig. 5*C*), while PC(16:0/16:0) and LPC(16:0) did not, as shown by a dose-dependency experiment (Fig. 5, *D*–*E*).

Primary cortical neuronal cultures were treated with different doses of PC(16:0/16:0) and LPC(16:0) followed by CCK8-based viability assay to demonstrate if PC(16:0/16:0) and LPC(16:0) are toxic to cultured neurons. As shown in Figure 5*F*, LPC(16:0) caused a significant increase in cell death (∗∗*p* < 0.01 and ∗∗∗*p* < 0.001 compared with DMSO treated controls group) at a level similar to that by the glutamate toxicity, which serves as a positive control. In contrast, PC(16:0/16:0) was not toxic to cultured neurons (Fig. 5*G*).

### LPC(16:0) induces significant intracellular calcium influx in neurons

**Figure 6:**
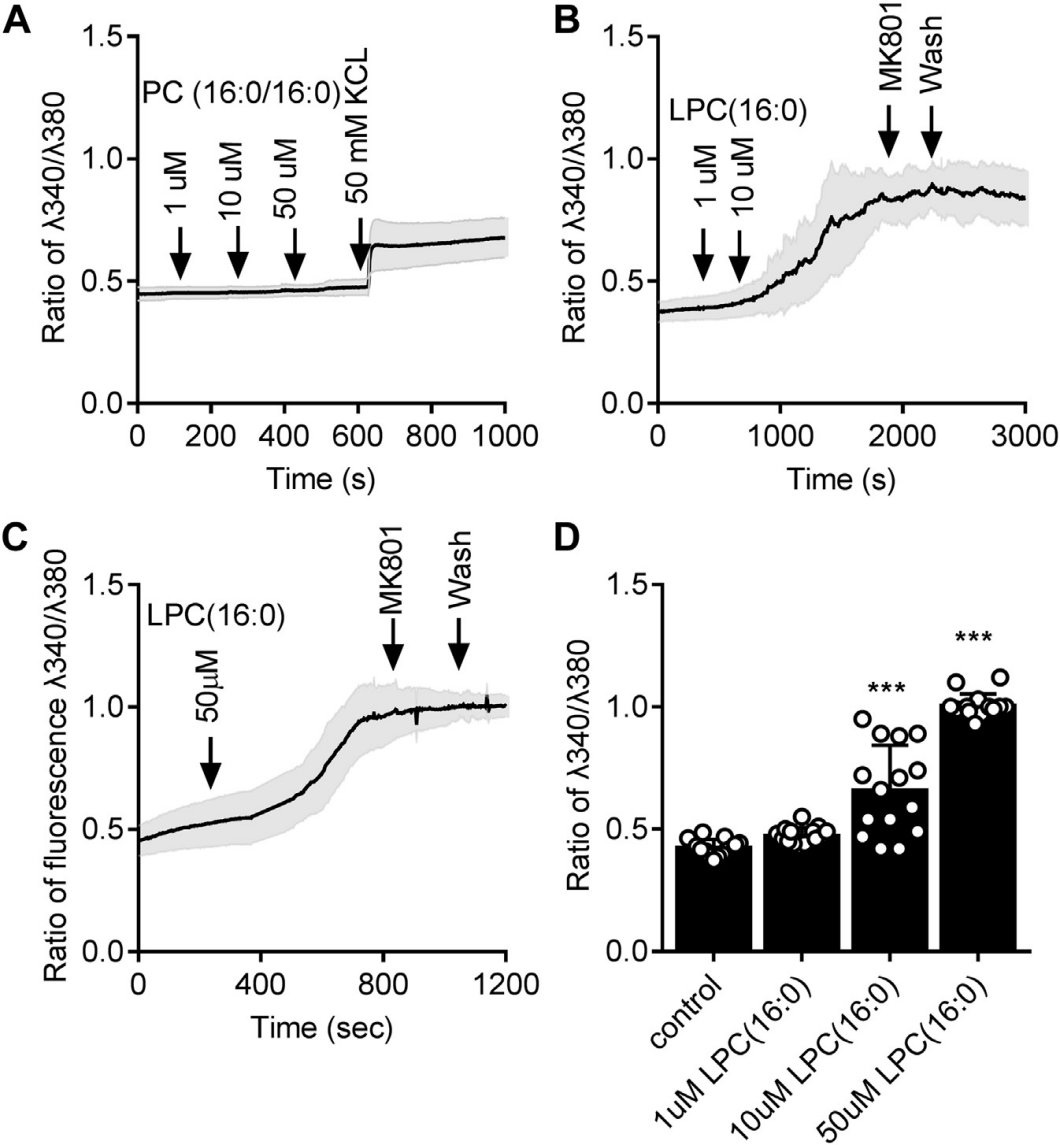
**Intracellular calcium influx in neurons in response to LPC(16:0). Cultured cortical neurons were loaded with Fura-2 for calcium imaging.***A*, treatment with PC(16:0/16:0) at the indicated concentrations did not elicit an increase in [Ca^2+^]i. KCl at 50 mM increased [Ca^2+^]i to show membrane potential changes. In contrast, LPC(16:0) at the indicated concentrations clearly increased [Ca^2+^]i at 10 μM (*B*), and 50 μM (*C*). Glutamate receptor inhibitor MK801 failed to block lipid LPC-induced calcium influx. The peak level of [Ca^2+^]i was quantified, as shown in *D*. Data represents the mean ± SD. Error bars indicate SD. n = 5 independent repeats, ∗∗∗*p* < 0.001 by one-way ANOVA with LSD *post hoc* analysis to identify significant groups.

To shed light on the possible mechanism of LPC(16:0)-induced toxicity to neurons, cultured cortical neurons were subjected to measurement of intracellular calcium influx [Ca^2+^]i (Fig. 6). PC(16:0/16:0) at concentrations of 1 μM, 10 μM, and 50 μM did not cause measurable increase in [Ca^2+^]i (Fig. 6*A*). In contrast, LPC(16:0) at 10 μM and 50 μM concentrations caused a significant increase in neuronal [Ca^2+^]i (Fig. 6, *B*–*D*, ∗∗∗*p* < 0.001 compared with the control group). LPC(16:0)-induced [Ca^2+^]i was independent on glutamate receptor activity as MK801, a competitive glutamate receptor antagonist, failed to block LPC(16:0)-induced [Ca^2+^]i.

### PC(16:0/16:0) suppresses microglial production of IL-1β and TNF-α

**Figure 7:**
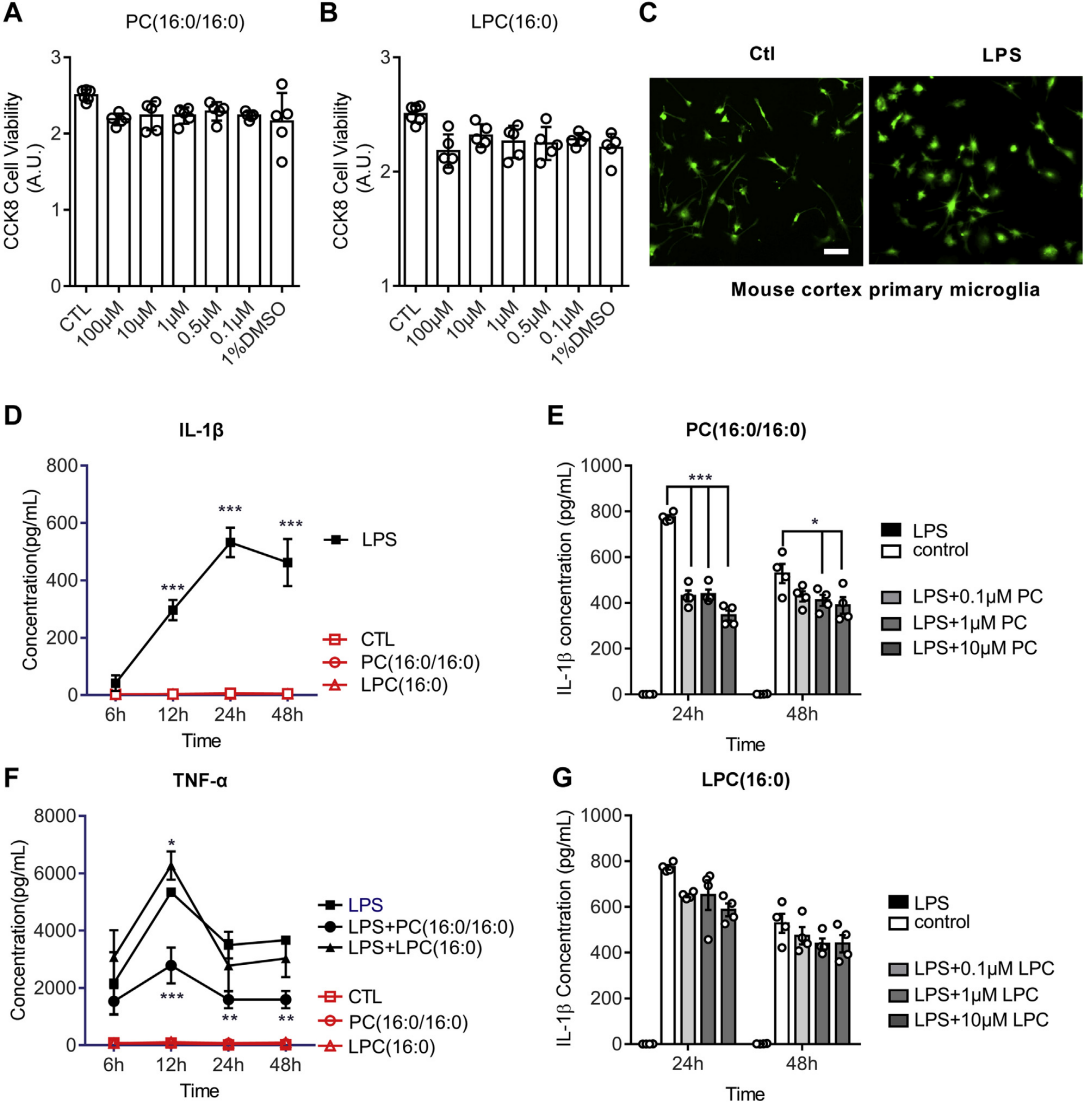
**Inhibition of microglial activation by PC(16:0/16:0).** Cultured mouse brain primary microglia were treated with PC(16:0/16:0) (*A*) and LPC(16:0) (*B*) at the indicated concentrations. Cellular toxicity was assessed using the CCK-8 viability kit. *C*, micrographs showing primary microglia untreated (left panel) and LPS-treated (right panel), followed by immunohistochemical staining with Iba1 antibody. The scale bar = 20 μm. ELISA assay was performed to show LPS-induced significant expression of IL-1β in cultured microglia (*D*), but not by LPC(16:0) and PC(16:0/16:0) treatments. Adding LPS with PC(16:0/16:0) at the indicated dosage significantly inhibited LPS-induced IL-1β expression (*E*), but not with LPC(16:0) (*G*). Similarly, PC(16:0/16:0) also significantly inhibited LPS-induced induction of TNK-α, but not by LPC(16:0). Data represent the mean ± SD. Error bars indicate SD. n = 5 independent repeats; ∗ indicates *p* < 0.05, ∗∗*p* < 0.01, ∗∗∗*p* < 0.001 by one-way ANOVA with LSD post hoc analysis to identify significant groups.

Mouse primary microglia were isolated and cultured, as previously described (28, 38). Treatment of cultured microglia with PC(16:0/16:0) and LPC(16:0) did not cause visible toxicity to cultured cells (Fig. 7, *A*–*B*) as determined using the CCK8 assay kit. Cultured microglia can be activated with the treatment of lipopolysaccharides (LPS) at 10 μM as shown in Figure 7*C*. Immunostaining of activated microglia with Iba1, a marker for microglia, showed an altered microglia morphology (Fig. 7*C*). LPS treatment also elicited an increased secretion of IL-1β (Fig. 7*D*) and TNF-α (Fig. 7*F*) as determined by the ELISA assay. Treatment of cultured microglia with PC(16:0/16:0) or LPC(16:0) at the indicated doses did not induce the secretion of IL-1β (Fig. 7*D*, red-colored lines) or TNF-α (Fig. 7*F*, red-colored lines). However, PC(16:0/16:0) (Fig. 7*E*), but not LPC(16:0) (Fig. 7*G*), significantly suppressed the LPS-induced IL-1β production. Collectively, these data strongly suggest that PC(16:0/16:0) may modulate the inflammatory response, while LPC(16:0) induces cytotoxicity in the recovering ischemic brain.

## Discussion

The present study systemically and semiquantitively investigated the profile of lipids in the ischemic mouse brain during long-term recovery. This unbiased shotgun approach allowed us to identify 84 differentially expressed lipid spices belonging to 15 lipid groups. The first 7-day post-MCAO represents a critical time window with the most upheaval of lipid changes, such as PE, DG, TG, and PC. In comparison, lipids at the 14 and 28 days post-MCAO returned to almost normal levels. Our data confirmed previously reported lipid changes following cerebral ischemia, such as LPC and Cer. Importantly, through the determination of the highly diverse and asymmetrically distributed fatty acid chains in phospholipids, we were able to show, for the first time, that the imbalanced ratio between PC(16:0/16:0) and LPC(16:0), controlled by the Lands cycle, was involved in cerebral ischemia. Indeed, ATK, an inhibitor to cPLA2, effectively prevented MCAO evoked imbalance of the PC-LPC ratio. These data shed light on the complexity of post-stroke brain lipid metabolism and indicated a future research direction to understand better the Lands cycle in ischemia brain injury and recovery.

Studies in a rat model of transient MCAO showed that LPC level increased dramatically in the rat brain following 5 min of focal cerebral ischemia, which returned to normal after 30 min reperfusion (23). A more recent study using 1 h occlusion time followed by 2 h reperfusion in mouse also showed increased LPC contents (39). This short-term change in LPC reported in the rodent models is different from what we described here. We found an early and sustained increase in LPC(16:0) in the ischemic brain for up to 7 days post-MCAO. The possible explanations for this discrepancy may come from the difference in the rodent models used in the studies and the lack of a precise determination of the LPC fatty acid chain length reported in the rat model. Moreover, our study focused more on the long-term and sustained lipids changes during reperfusion rather than short-term and transient changes, which may underlie the discrepancies between our findings and the reports in the literature.

Brain phospholipids are highly enriched with long-chain polyunsaturated fatty acids. Among all 84 differentially expressed lipids, almost all of them are long-chain polyunsaturated fatty acid chains (Table 1), except PC(14:0/16:0), PC(16:0/12:0), PC(16:0/13:0), PC(16:0/16:0), LPC(16:0), and LPE(16:0). These saturated fatty acids accounted for only about 7% of the 84 lipids. Of the six lipids we were able to quantify, the lipid contents in the Sham brain are ranked from high to low as PE(16:0/18:1) > LPC (16:0) > Cer(d18:1/17:0) > PC (16:0/16:0) > LPE(16:0) > LPE(18:1). The dramatic increase in the level of PE(16:0/18:1) to 22 mg/g 3 days after MCAO, from a previous 8.8 mg/g 1 day after MCAO, was interesting. However, the role of PE in general, and PE(16:0/18:1) in particular, remains virtually unknown in the ischemic brain.

Based on the understanding that knockout and inhibition of cPLA2 conferred neuroprotection against ischemic injury (25, 26), it is a plausible hypothesis that the Lands cycle is involved in cerebral-ischemia-induced brain injury. Overstimulation of cPLA2 leads to the breakdown of membrane PC and subsequent accumulation of LPC in the damaged tissue. Indeed, cPLA2-specific inhibitor ATK successfully inhibited the flux of conversion from PC(16:0/16:0) to LPC(16:0) during the recovery of MCAO, as shown in Figure 4*F*, which strongly supports the role of the Lands cycle in the ischemic injury.

LPC has been shown to be secreted from apoptotic cells, which in turn plays a role in the inflammatory reaction mediated by microglia (40). Indeed, the present study showed that PC(16:0/16:0) suppressed LPS-evoked microglial production of IL-1β and TNF-α, while LPC(16:0) did not, indicating that cerebral ischemia tipped the balance of PC-LPC ratio, which is regulated by the Lands cycle. LPC(16:0) also caused significant neuronal growth cone collapse and toxicity in cultured cortical neurons, confirming previous reports that increased LPC in the injured brain is detrimental to brain recovery (41, 42). The mechanisms of LPC toxicity can be complex. Lysolipids and fatty acids form micelles in solution and act as detergents in the presence of lipid membranes (43). As such, lysolipids can increase ion permeability (44) and modify membrane channel function (43, 45). Indeed, inhibition of LPC acyltransferase downregulates inflammatory cytokine production in monocytes and epithelial cells by preventing translocation of Toll-like receptor 4 into membrane lipid rafts to regulate the inflammatory response (46).

Together, the current study demonstrated that the PC(16:0/16:0) and LPC(16:0) ratio maintained by the Lands cycle is important during the development of cerebral ischemic brain injury. Future studies in the animal brain using molecular genetics approaches targeting Lands’ cycle may provide novel insights into developing therapeutics against lipid-induced inhibition of brain recovery from stroke.

## Experimental procedures

### Transient MCAO surgery and ARRIVE guidelines

All procedures using animals were approved by the Animal Care Committee of the Southern University of Science and Technology. The ARRIVE guideline was followed when designing and performing animal experimentation (47). Efforts were made to minimize the number of mice used. The animal inclusion criterion was based on the exact age, sex, and bodyweight of the mice. The exclusion criterion was when the mouse failed to survive MCAO surgery at the end of the 28-day periods of reperfusion. A minimal of five mice per group were used as indicated in the figure legends to achieve meaningful statistical differences. A total of 30 mice with the same age (3-month-old), sex (male), and body weight (23 g) were randomly assigned to two groups to receive Sham (5 mice) or MCAO (25 mice) surgeries, respectively.

C57BL/6 mice (20–23 g) were obtained from the Guangdong Provincial Animal Facility (Guangzhou, China). The eGFP transgenic mice (C57BL/6-Tg(CAG-EGFP)C14-Y01-FM131Osb) were gifts from the SUSTech Animal Core Facility inventory. Mice were maintained in an SPF II animal facility with a controlled environment (temperature: 22 °C ± 1 deg. C; humidity: 50 ± 10%, and a 12 h light/dark cycle). Mice were allowed food and water *ad libitum*. Experimenters were blinded to animals’ treatments and sample processing throughout the subsequent experimentation and analyses.

Under temporary isoflurane anesthesia, mice were subjected to 30 min MCAO using an intraluminal filament, as previously described (48, 49). After 30 min of MCAO, the filament was withdrawn, and blood flow was restored to basal levels, as assessed by laser Doppler flowmetry. Wounds were sutured following surgery. The body temperatures of experimental mice were monitored before and after the MCAO surgery using a rectal probe and were maintained at 37 °C utilizing a heating pad and a lamp. In preliminary experiments to verify a consistent stroke procedure, measurements of blood pressure, blood gases, and pH were also performed. Sham-operated mice were subjected to the same surgery without MCAO and used as controls.

Arachidonyl trifluoromethyl ketone (ATK), a potent inhibitor of cPLA2, was purchased from Cayman Chemicals (MI, USA). Freshly prepared ATK (7.5 mg/kg body weight) with a 0.5% methylcellulose (Sigma-Aldrich, St Louis, MO, USA) carrier solution was gavage fed to mice 2 h after the MCAO surgery and every 24 h thereafter until the end of the experiment. Brain cortices were collected for PC and LPC determination using MS/MS.

### Measurements infarct size

Infarct size was measured by a colorimetric staining method using 2,3,5-triphenyl tetrazolium chloride (TTC) as described previously (28, 50). Briefly, brains were dissected out and cut into four 2 mm thick coronal slices, which were stained with 5 ml of 2% TTC for 90 min at 37 °C. Brain slices were imaged using a photo scanner (HP ScanjetG4010). The infarct size was measured using Image J based on the measurements of the volume of the white-colored areas in the ipsilateral side of the brain x the length of the brain slices. The edema volume was taken away to derive at the final ipsilateral side of the infarction. The ratio of brain infarction was calculated as exactly previously described (50).

### Neurological deficit scores

A six-point scale assessment and forelimb grip strength test were performed exactly as previously described (48, 49). (a) An expanded six-point scale turning behavior test: Assessments were made by an individual blinded to the treatment of the mice 30 min after MCAO when animals were fully awake after anesthesia. The neurological deficits were scored as follows: 0, normal; 1, mild turning behavior with or without inconsistent curling when picked up by the tail, 50% attempts to curl to the contralateral side; 2, mild, consistent curling, 50% attempts to curl to contralateral side; 3, strong and immediate consistent curling, mouse holds the curled position for more than 1 to 2 s, the nose of the mouse almost reaches the tail; 4, severe curling progressing into barreling, loss of walking or righting reflex; 5, comatose or moribund. (b) The forelimb grip strength test was performed using the Grip Strength Meter from Columbus Instruments (MyNeurolab, St Louis, MO), which measures forepaw muscle strength and neuromuscular integration relating to the grasping reflex in the forepaws. The peak preamplifier automatically stores the peak pull force and shows it on a liquid crystal display. For each animal, at least ten measurements were taken at a specific time point, and the mean and standard error were calculated.

### Brain sampling and lipid extraction

After MCAO/reperfusion, mice were killed at the specified dates. The brain was quickly dissected out and placed on ice. The two cerebral cortices were peeled off and collected for lipid extraction. Brain samples were stored at −80 °C and then thawed at 4 °C before lipid extraction. The extraction method was as previously described (51). Briefly, brains were homogenized in 120 μl precooled isopropanol, vortexed for 1 min, and incubated for 10 min at room temperature. The mixture was stored overnight at −20 °C to improve protein precipitation. Samples were centrifuged for 20 min at 14,000*g*, and then the supernatant was further diluted with IPA/acetonitrile (ACN)/H2O (2:1:1 v:v:v) and stored at −80 °C until LC-MS analysis. Equal amounts of all samples were pooled as a QC sample for LC-MS system conditioning and quality control.

### UPLC-MS/MS

Lipidomics was performed at SUSTech Proteomics Platform Core Facility, utilizing the Thermo Scientific Q Exactive hybrid quadrupole-Orbitrap mass spectrometer. Chromatographic separation was performed with a Thermo BEH UPLC C18 column (2.1 × 100 mm, 1.7 μm). Mobile phase A consisted of 10 mM of ammonium formate and 0.1% formic acid (ACN: H_2_O = 3:2, v/v with 10 mM NH_4_AC), and mobile phase B consisted of 10 mM of ammonium formate and 0.1% formic acid (IPA: ACN = 9:1, v/v with 10 mM NH_4_AC). A flow rate of 0.4 ml/min was used. The elution gradient used were at 0 to 1.5 min with 32 to 32% B; 1.5 to 15.5 min with 32% to 85% B; 15.5∼15.6 min with 85% to 97% B; 15.6 to 18 min with 97% to 97% B; 18.0∼18.1 min with 97%∼32% B; 18.1 to 22.0 min with 32% to 32% B. Both positive and negative modes were performed with an acquisition time of 0.2 s per scan. The scan range was set at 133.3 to 2000 Da. The capillary was set at 4.5 kV and −3.5 kV in positive ion mode and negative ion mode, respectively. The capillary temperature was set to 350 °C. Furthermore, quality control samples were spiked in experimental samples to evaluate the stability of the LC-MS system during acquisition.

### Lipidomics data analysis

The raw LC-MS files were imported into Lipidsearch software (Thermo Fisher Scientific, USA) for peak alignment and picking. Data generated were further preprocessed using Xcalibur 4.0 software (Thermo Fisher Scientific). Lipids were removed from further analysis if they were less than 50% of the QC samples [SM(d18:1/12:0)] or less than 20% of the experimental samples. Data were then imported into Simca-p 14.0 for PCA and orthogonal partial least squares discriminant analysis (OPLS-DA) to validate the lipidomic data in terms of stability and reliability between groups and to provide insights into separations between experimental groups based on high-dimensional spectral measurements.

### Primary cortical neuron and explant cultures

Primary cortical neuron culture was performed as previously described (52, 53). Briefly, the E15 embryonic mouse cerebral cortex was treated with papain (P3125, Sigma-Aldrich) dissolved in Neurobasal Media (Gibco, USA) for 30 min at 37 °C to dissociate cortices into single cells. After resuspension in neurobasal media containing trypsin inhibitor, tissue chunks not sufficiently digested were removed using brief centrifugation. Fully separated cortical neurons in the supernatant were collected and plated at 5 × 10^4^ cells/ml density. Neurons were cultured at 37 °C in a humidified atmosphere of 5% CO_2_ for 7 to 8 days before use.

Cortical explants were also made from E15 embryonic mouse cerebral cortex and cultured for 3 to 7 days as described for the cortical neuron cultures above. After treatments, the culture medium was removed, and the cells were fixed in freshly prepared 4% paraformaldehyde (in 1× PBS) for 20 min. Cells were washed in PBS twice, 5 min each, incubated with blocking solution (1.5% BSA in PBS, containing 0.2% Triton X-100 and 0.02% NaN3) for 30 min, and stained with rhodamine-labeled phalloidin (ab235138, Abcam, USA; 1:100, in blocking solution) for 1 h. After washing with PBS (3 min each for 3 times), deionized water (once), cells were mounted in Dako mounting medium (spiked with Hoechst 32558) and dried for microscopic analysis. A Sholl method (37) was used to determine PC(16:0/16:0) and LPC(16:0) effects on neurite outgrowth. Neurite lengths on the digitized images were measured using Image J, an image analysis system (NIH Image J software located at http://rsb.info.nih.gov/ij/).

### Primary mouse microglia cultures

Primary mouse microglia was cultured to a greater than 98% purity from the neonatal mouse brain using the exact method as previously described (28, 38). Briefly, mixed glial cultures were prepared from the cerebellum of 6 to 9 day-old neonatal mice. Cells were maintained in Dulbecco’s Modified Eagle Medium (DMEM)/F12 (GIBCO, Cat:11330) with 10% FBS (Hyclone) at 37 °C in a humidified incubator with 5% CO_2_. The medium was replaced every 4 to 5 days until cells reached confluence. Trypsin solution (0.25% Trypsin, 1 mM EDTA) was diluted 1:4 in DMEM/F12. The culture medium was removed, and the diluted trypsin solution was placed. The detached upper layer cells were removed after incubation for 30 min at 37 °C. Trypsin was inhibited by adding 10% of serum, and the detached upper layer cells and trypsin solution were removed. The majority (>98%) of cells left behind and that were still attached to the plate were microglia. The isolated microglia were maintained in DMEM-F12 with 10% FBS until use.

### Ratiometric measurement of [Ca^2+^]i using Fura-2

Ratiometric calcium imaging was performed on neurons to detect intracellular calcium levels, as we have previously described (48). Briefly, cortical neurons on coverslips in 24 well plates were treated with Fura-2-AM fluorescence dye (molecular probes, Life Technologies, USA) in a final concentration of 5 μl/ml and incubated at 37 °C with 5% CO_2_ for 20 min. After rinsing twice with 37 °C PBS Mg^2+^-free buffer, the coverslips were transferred into a new 35 mm dish containing 3 ml PBS Mg^2+^-free buffer. Fura-2 fluorescence was measured at 510 nm emission with 340/380 nm dual excitation selected by a DG-5 system (Sutter Instrument Company, Novato, CA) and imaged with a fluorescence microscope (Leica DMI4000B, Germany). The intracellular calcium ([Ca^2+^]i) concentration was expressed as ratios from fluorescence intensity between the two excitation wavelengths of R340/380 of Fura-2. Mg^2+^-free buffer containing 45 mM KCl was applied to cells to induce [Ca^2+^]i in order to confirm neuronal membrane potential and viability following all treatments. All measurements were independently repeated at least three times. For each experiment, at least 10 cells were selected for analysis.

### Neuronal viability assay using CCK8 kit

Neuronal viability was assessed with a cell viability counting kit CCK-8 purchased from BBI Life Sciences (Shanghai, China). Briefly, cells after treatment were collected following the manufacturer’s protocol. Cell suspension at 100 μl was transferred to a 96-well plate at a final concentration of 500,000 cells/ml. Cells were incubated with 10 μl CCK-8 solution from the kit per well for 4 h and assessed with a PerkinElmer spectrometer (PerkinElmer, USA) at the absorbance of 450 nm.

### ELISA

To detect the secretion levels of IL-1β and TNF-α in mice cerebral cortexes, mice cerebral cortexes were dissected out and homogenized in cell lysis buffer supplemented with phenyl-methyl-sulphonyl fluoride. After centrifugation, the supernatants were used for ELISA according to the manufacturer’s protocol (R&D Systems, USA). Optical densities were measured using a Model 680 microplate reader (Bio-RAD, Hercules, CA) at 450 nm.

### Statistical analysis

All data were represented as mean ± SD. The nonparametric Spearman’s rank-order correlation coefficient was used to determine the strength and direction of the monotonic relationship between PC and LPC content variables. A comparison of lipidomics data was performed using Student’s *t*-test. One-way ANOVA plus LSD *post hoc* analysis was employed to analyze MCAO data, the neuronal viability, and ratiometric measurement of [Ca^2+^]i data analyses. All analyses were performed using GraphPad Prism 7.0 software. A *p* value <0.05 was taken to indicate statistical significance.

## Data availability

The data generated and analyzed in this study can be obtained from the authors upon reasonable request.

## Conflict of interest

The authors declare that they have no conflicts of interest with the contents of this article.
